# Mapping Scalp to Intracranial EEG using Generative Adversarial Networks for Automatically Detecting Interictal Epileptiform Discharges

**DOI:** 10.1109/SSP53291.2023.10207965

**Published:** 2023-07-02

**Authors:** Bahman Abdi-Sargezeh, Ashwini Oswal, Saeid Sanei

**Affiliations:** * Department of Computer Science, Nottingham Trent University, Nottingham, UK; † Nuffield Department of Clinical Neurosciences, University of Oxford, Oxford, UK

**Keywords:** Generative adversarial network, IED detection, interictal epileptiform discharges, epilepsy mapping scalp to intracranial EEG

## Abstract

Both scalp and intracranial electroencephalograms (EEGs) are of great importance for diagnosing brain disorders. However, the scalp EEG (sEEG) is attenuated by the skull and contaminated with artifacts. At the same time, intracranial EEG (iEEG) is almost free of artifacts and can capture all brain activities without any attenuation due to being close to the brain sources. In this study, the aim is to enhance the performance of sEEG by mapping the sEEG to the iEEG. To do so, we here develop a deep neural network using a generative adversarial network to estimate the sEEG from the iEEG. The proposed method is applied to sEEG and iEEG recorded simultaneously from epileptics to detect interictal epileptiform discharges (IEDs). The proposed method detects IEDs with 76% accuracy outperforming the state-of-the-art methods. Furthermore, it is at least twelve times less complex than the compared methods.

## I Introduction

The electroencephalogram (EEG) is a recording modality that captures the brain electric activity. It can be recorded from the scalp, cerebral cortex, or deeper brain [1]. The scalp EEG (sEEG) is non-invasive and easy to record; however, it is contaminated with noise and unable to capture weak activities of deep brain sources. On the other hand, the intracranial EEG (iEEG) channels, inserted through foramen ovale (FO) holes, hence called FO electrodes, record the brain activities clearly with a small amount of artifact. However, the problem with iEEG recordings is that they are recorded using invasive techniques, which involve hazards for the patients. Therefore, we aim to recover the iEEG details from the concurrent sEEG by mapping the sEEG to the iEEG using developing a deep network based on generative adversarial networks (GANs).

GANs comprise a generator network and a discriminator network [2]. The generator network generates data from a latent space or the observed data sample. The discriminator network discriminates the real data from the generated data. They have been extensively used for mapping speech-to-image [3], speech-to-speech [4], and particularly image-to-image [5]. In the EEG signal processing area, GANs are mostly used for data augmentation [6]–[8] as the collection of a large amount of EEG data is not only time-consuming but also costly. In [8], the authors developed a GAN for generating augmented spike and non-spike EEG signals, then employed a deep network to classify spikes and non-spikes. Recently, a GAN architecture was proposed to synthesize the visual stimulus shown during EEG recording from EEG signals [9]. Unlike previous studies, we aim to design a GAN to map the scalp to intracranial EEG. Hence, it is referred to as EEG-to-EEG translation.

We aim to apply our model to the concurrent sEEG and iEEG signals recorded from patients suffering from epilepsy. The signals include interictal epileptiform discharges (IEDs), transient activities occurring between two seizure onsets [10]. In terms of morphology, IEDs appear in spikes, poly spikes, or sharp waves, followed by slow waves [11]. The IEDs are generated using deep sources in the brain. Therefore, iEEG recordings are mainly used to capture the IED signature. On the other hand, the sEEG fails in capturing most IED signatures because of being far away from IED sources and attenuated by the skull. Only 9% [12] to 22% [13] of IEDs were observed in sEEG recordings in previous studies in which the sEEG and the iEEG were recorded and analyzed simultaneously. This means that a large proportion of IEDs are invisible over the scalp.

In most studies, IEDs are detected either from sEEG [14] or iEEG [15]. Studies employing their methods to detect IEDs from only sEEG recordings are limited to detecting only scalp-visible IEDs. To overcome this limitation, our research group developed methods to detect both scalp-visible and scalp-invisible IEDs from the sEEG [16]–[20]. These studies employed concurrent sEEG and iEEG recordings. The iEEG was used as the ground truth to annotate IEDs, then the IEDs were detected from the sEEG.

We have already mapped the time-frequency features of sEEG to those of iEEG using the developed tensor factorization technique [20]. We also developed two methods based on autoencoder (AE) for mapping sEEG to iEEG [21]. Here, we aim to develop a deep learning method using a GAN structure to map sEEG to iEEG.

## II EEG-TO-EEG Translation

Let X ∈ ℝ^64×12^ and Y ∈ ℝ^64×12^ be respectively the sEEG and iEEG, where 64 and 12 are respectively the number of time samples and channels. The aim is to design a learning system and apply sEEG **X** to generate an estimation of iEEG **Ỹ**. The network is designed based on GAN.

### A Objective

A GAN consists of a generator network G and a discriminator network D. The generator G is fed with the sEEG **X** to generate an estimation of iEEG, $\tilde{Y}=G(X)\text{.}$ The discriminator D takes either the concatenation of the sEEG and the iEEG **Y** (referred to as real) or the concatenation of the sEEG and the estimated iEEG (referred to as fake) as input and predicts a binary class of real or fake. An adversarial loss is employed to train the generator and discriminator. Like most studies [5], [22] including the original GAN [2], the binary cross entropy is used in a min-max game approach according to the following loss function:

(1) $$\begin{array}{lll} \underset{G}{\min}\underset{D}{\max}L_{GAN}(G,D) & = & E_{\left(X,Y\right)}[\log(D(X,Y))]+\\ & & E_{X}[log(1-D(X,G(X)))], \end{array}$$

where G minimizes the objective loss function against an adversarial D maximizing it.

To have a more accurate estimation, we regularize the GAN objective function with *L*_2_ distance (norm), estimated as follows:

(2) $$L_{2}=E_{\left(X,Y\right)}[∥Y-G(X){∥}_{2}].$$

The discriminator network remains unchanged, but the generator loss is coupled with *L*_2_ distance and applied to train the generator:

(3) $$L_{G}=\underset{G}{\min}\underset{D}{\max}L_{GAN}(G,D)+\lambda L_{2}$$

where *λ* is the coefficient of *L*_2_ loss function.

### B Generator

**Fig. 1:**
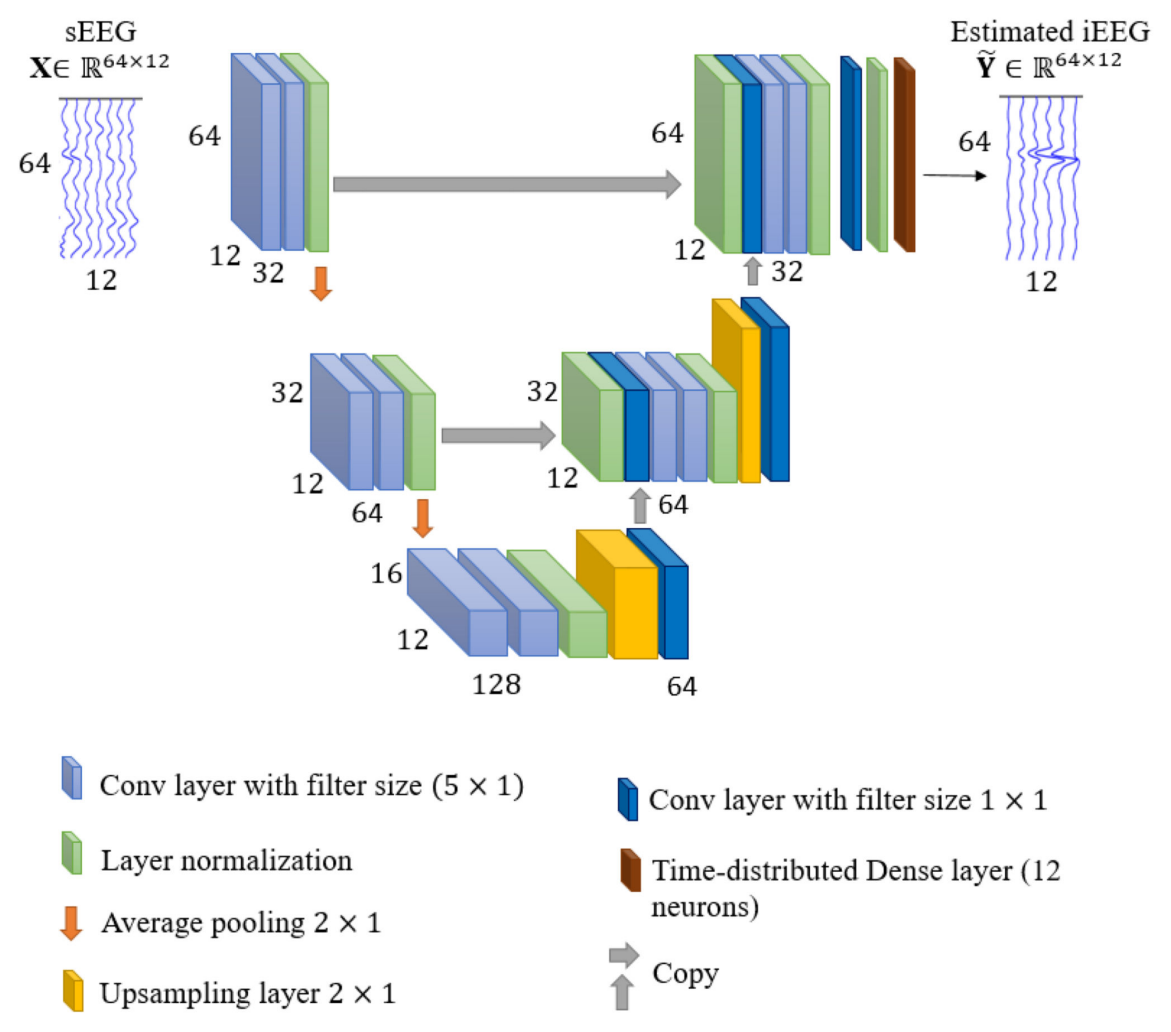
The generator network architecture.

Our generator network is designed based on a U network (U-net). The architecture of the generator is shown in Fig. 1. It is made up of a contracting path (left side) and an expansive path (right side). The contracting path consists of repeated convolutional layers with the filter size of 5 × 1, each followed by a normalization layer operation across time domain and an average pooling operation with stride 2 × 1 for downsampling. At each downsampling step we double the number of feature channels.

Every step in the expansive path consists of an upsampling of the feature map with the size of 2 × 1 performed bilinearly, a 1 × 1 convolution, a concatenation with the corresponding feature map from the contracting path, two 5 × 1 convolutions, and a normalization layer operation across time domain. The output of final expanding layer is fed to a 1 × 1 convolutional layer. Finally, a time distributed dense layer is employed to map features of each time component to 12 neurons (the same number as iEEG channels).

Here, we set the number of scalp channels the same as that of intracranial channels since the sEEG is concatenated with the iEEG to be fed to the discriminator. We selected scalp channels from temporal and frontal areas since the IEDs origin from these brain regions.

### C Discriminator

**Fig. 2:**
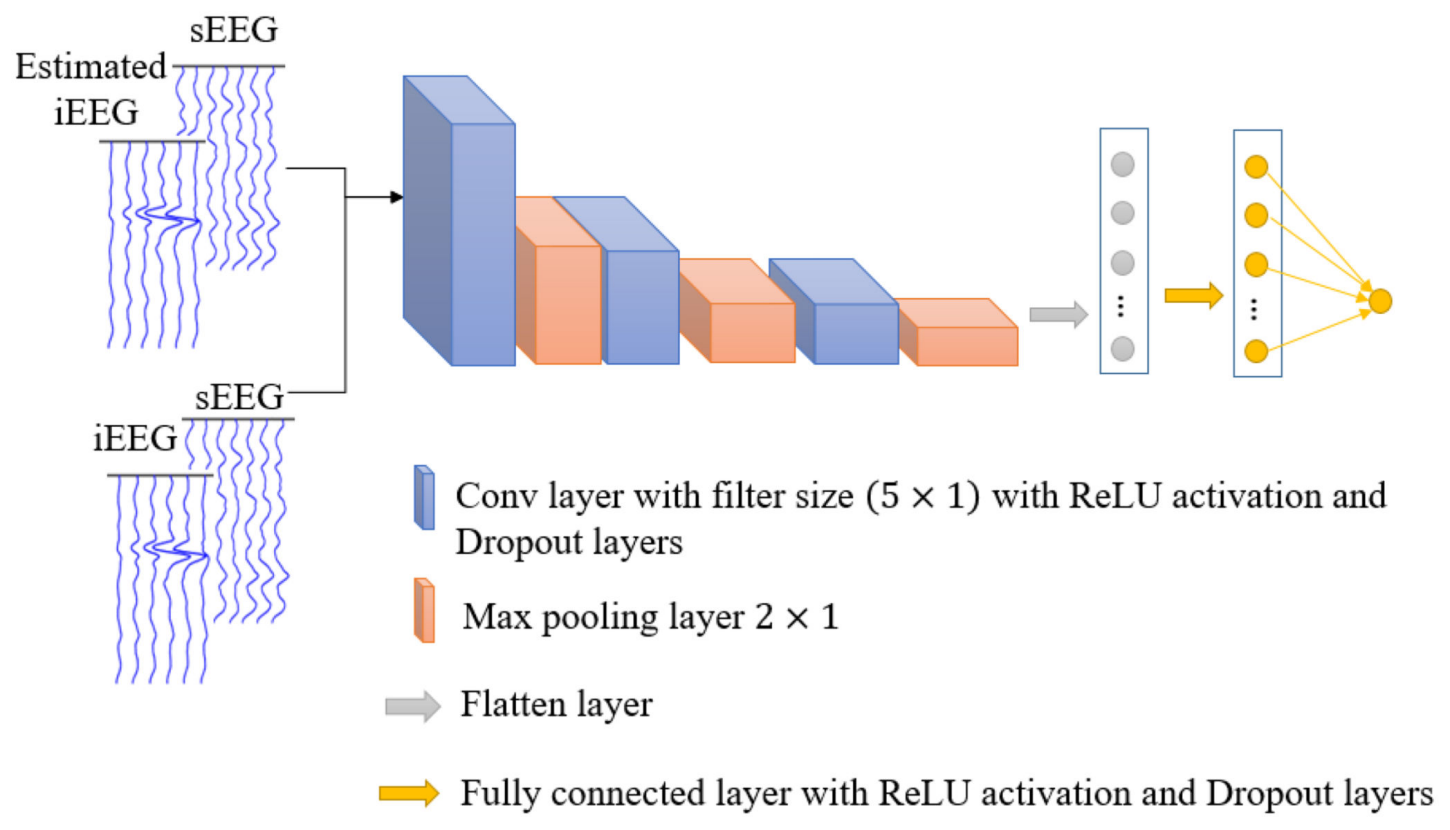
The discriminator network architecture.

Our discriminator follows the typical architecture of a convolutional network, shown in Fig. 2. It consists of 5×1 convolutional layers – each of which is followed by a ReLU activation layer, a dropout layer, and a max pooling layer with the size of 2 × 1 – and a fully connected dense layer. The input of the discriminator is the concatenation of sEEG with either the estimated or real iEEG. The effectiveness of this concatenation technique has been proven in mapping studies [4], [5].

## III Experiment

We applied our proposed GAN to estimate iEEG from sEEG (mapping sEEG to iEEG). Then, the IEDs were detected from the estimated iEEG.

### A Dataset

The sEEG and iEEG signals of 18 epileptic subjects were simultaneously recorded at King’s College Hospital London. The signals were recorded at a sampling rate of 200 Hz. For recording the sEEG, 20 standard silver chloride electrodes were used, placed on the scalp according to the “Maudsley” electrode placement system. The iEEG were recorded by using 12 intracranial multicontact FO electrodes consisting of a couple of 6 electrode bundles. The FO electrodes were inserted through the patients’ FOs under general anaesthesia, fluoroscopic control and placed into the ambient cistern.

### B IED Annotation and Preprocessing

The iEEG was used as a ground truth to annotate IEDs. In other words, an expert neurologist labeled IEDs based on their morphology and spatial distribution observed in the iEEG. For mapping and classification, segment of 64 samples (32 samples before and after the spikes peak) were selected. We selected non-IED segments from where there was no sign of spikes. The number of IED and non-IED segments set equal. Different numbers of IEDs from 50 to 953 were annotated from each subject.

A bandpass filter with cutoff frequencies of 1 and 70 Hz as well as a notch filter with notch frequency of 50 Hz were applied to both sEEG and iEEG signals. In addition, common average reference was applied to the sEEG for re-referencing.

### C Mapping sEEG to iEEG

The sEEG is fed to the generator to generate an estimation of iEEG. Then, the concatenation of sEEG with either the real or estimated iEEG is fed to the discriminator to be classified respectively as real or fake. Because of the concatenation of sEEG with iEEG, the number of scalp and FO channels must be the same. Therefore, twelve out of twenty scalp channels are selected to be mapped to the iEEG. These 12 channels, namely Fp1, F3, F7, C3, T3, Fp2, F4, F8, C4, T4, Fz, and Cz, are selected from temporal and frontal regions, where the IEDs originate from.

### D Classification Network and Cross Validation

For detecting IEDs, the EEGNet [23] with minor changes is employed. The Batch normalization layers in EEGNet are eliminated in our network. In addition, instead of Average Pooling, we employ Max Pooling.

**Fig. 3:**
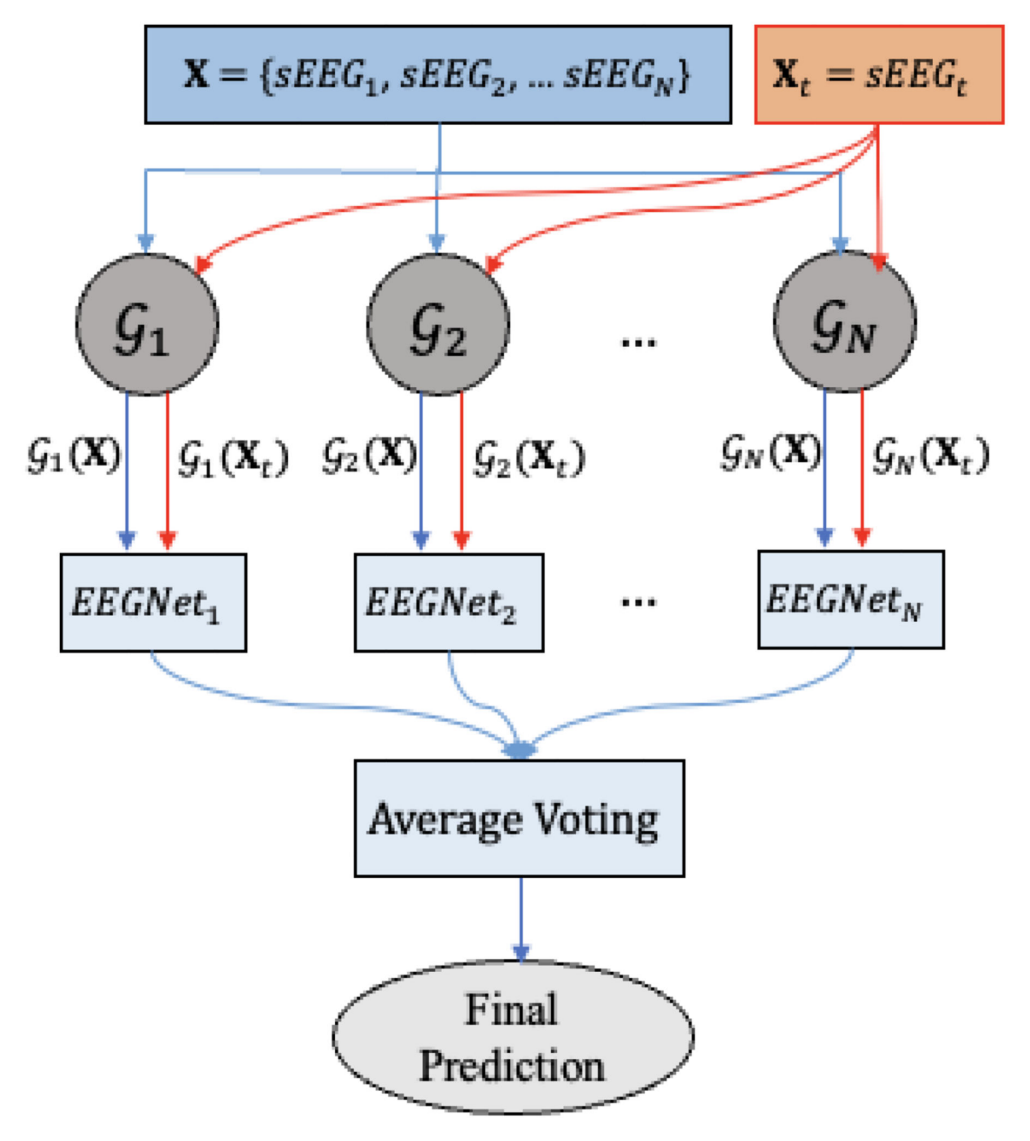
The diagram of the inter-subject classification approach.

The IEDs are detected in two different approaches: intra- and inter-subject classification approaches. In the intra-subject classification approach, the data of a subject is divided into training (70%), validation (10%), and test datasets (20%). In the inter-subject classification, the leave-one-subject-out cross validation is used. The data of *N* subjects are used for training the networks, and the data of one subject for testing. This approach is repeated for all 18 subjects. The sEEG of all *N* training and the test subjects are mapped to the iEEG using each of the trained GAN $\left(G_{n},n=\{1,2,\ldots ,N\}\right)$ to obtain an estimation of the corresponding iEEG, $X\overset{G_{n}}{\to }G_{n}(X)\text{.}$ Then, each of the estimated iEEG $G_{n}(X)$ is given to the EEGNet to differentiate IEDs and non-IEDs. Finally, to find the segment labels in the test data, the output probabilities of *N* EEGNets are averaged (average voting classification). Fig. 3 presents the diagram of the inter-subject classification approach. Here, the training data includes the data of *N* subjects whose IEDs are detected with high accuracy in the intra-subject classification approach.

## IV Results

We compare our developed methods with three widely referenced methods [21], [24]. In [24], a model developed based on least-squares regression (LSR) was employed to model the iEEG from sEEG recordings. A classifier trained with stepwise discriminant analysis was employed for classification. In [21], the authors first mapped the sEEG to iEEG by developing an asymmetric AE (AAE). They called it asymmetric since the number of input and output of AE were not the same. The output of AAE is called pseudo-iEEG. The pseudo- iEEG was again mapped to the real iEEG by feeding it to a symmetric AE. The overall method is called asymmetric-symmetric AE (ASAE). A convolutional neural network was employed for feature exploitation and classification. These methods are referred to respectively as AAE and ASAE. For evaluation, accuracy (ACC), sensitivity (SEN), specificity (SPC), and complexity of networks were estimated.

**Fig. 4:**
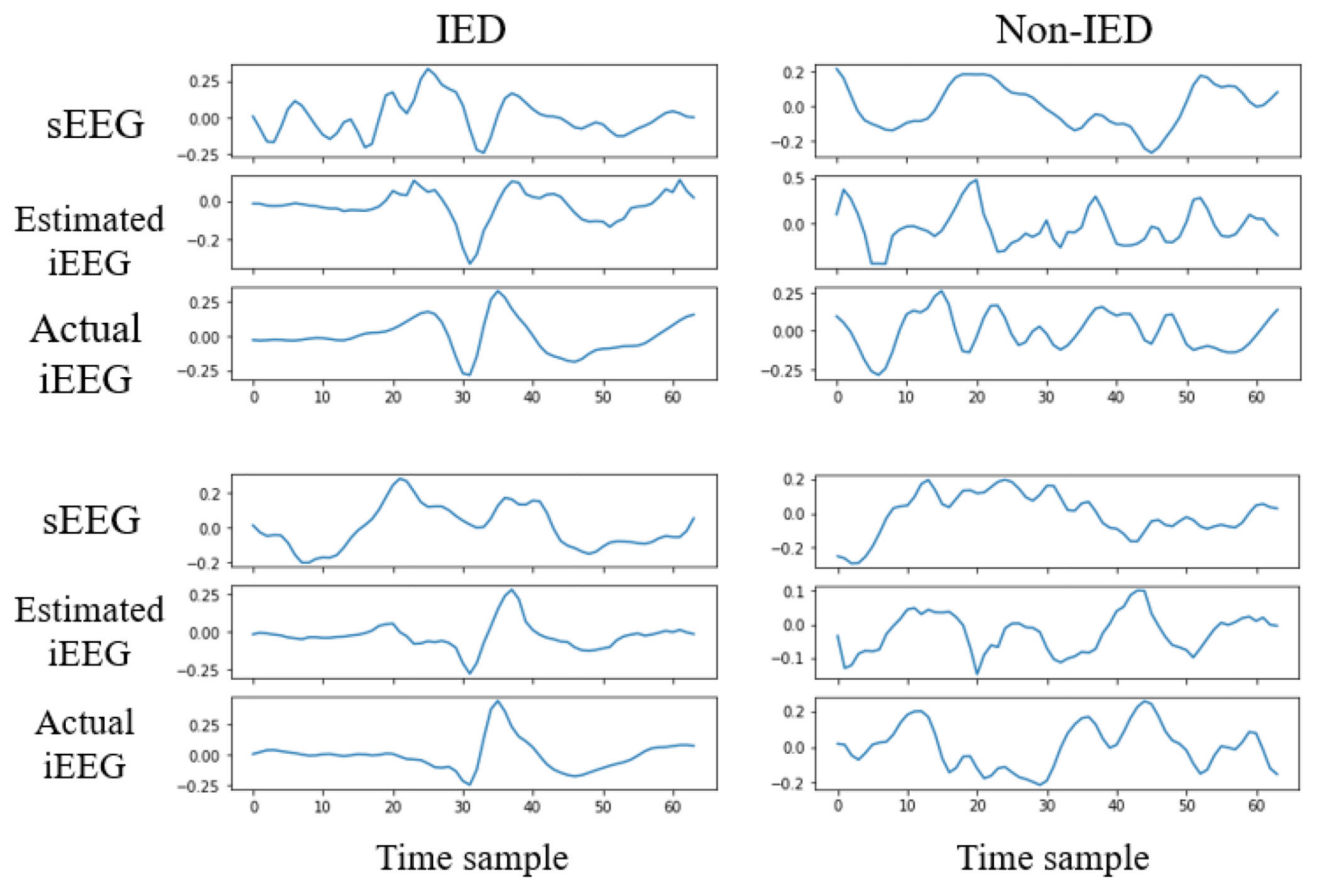
Samples of (a) IEDs and (b) non-IEDs. The sEEG, estimated iEEG (obtained using GAN), and actual iEEG are shown. The IEDs start at sample 32.

Fig. 4 shows a couple of IED and non-IED samples of sEEG, estimated iEEG, and actual iEEG. As it can be seen, the estimated iEEG precisely follows the trend of actual iEEG. This shows that our mapping model maps sEEG to iEEG with reasonable accuracy.

ACC presents how accurately the IEDs and non-IEDs are detected. The obtained ACC values are shown in Table I. Our proposed method outperforms the compared methods in the intra-subject classification approach by providing 76% accuracy. In the inter-subject classification approach, both GAN and ASAE achieve 68% accuracy which was respectively 2% and 6% more than accuracy values of AAE and LSR. Though GAN and ASAE provide the same accuracy value, in the ASAE structure two sequential mapping networks have been employed.

SEN shows the ability of a system in correctly detecting IEDs, while SPC shows the ability of a system in correctly detecting non-IEDs. The obtained SEN and SPC values are presented in Table II. ASAE achieves the best SEN of 67%. Our proposed GAN obtains the best SPC of 70% and detects IEDs with 65% SEN.

The last row in Table II shows the number of parameters of each model. Our proposed model is around respectively twelve and sixteen times less complex than AAE and ASAE. Having less complex networks is of great importance for online processing. This shows our proposed GAN is more effective and applicable than the compared methods.

## V Conclusion

Epilepsy diagnosis primarily relies on accurate detection of IEDs from sEEG. Only a small percentage of IEDs can be viewed from sEEG. Therefore, better highlighting these IEDs within sEEG is of great importance for seizure diagnosis. The proposed method here uses GAN to transfer a low resolution EEG to a high resolution counterpart to best highlight these IEDs. Our proposed method provides superior performance compared to the LSR, AAE, and ASAE approaches. It achieves the maximum accuracy values of 76% and 68% respectively in the intra- and inter-subject classification approaches. This is approximately four times the scalp-visible IEDs. Furthermore, our model is less complex than the compared methods.

## Figures and Tables

**Table I:** The ACC Values in the inter- and Intra-Subject Classification Approaches. The Amounts in Parenthesis Show the Intra-Subject Classification Performance. The Values are in Percent (%).

Subject	LSR [24]	AAE [21]	ASAE [21]	GAN
S1	65 (72)	85 (80)	87 (78)	67 (78)
S2	86 (81)	92 (82)	94 (88)	83 (95)
S3	65 (69)	72 (72)	69 (82)	74 (90)
S4	58 (62)	58 (71)	59 (77)	66 (81)
S5	55 (55)	64 (64)	65 (75)	67 (73)
S6	61 (59)	70 (60)	71 (63)	68 (68)
S7	59 (64)	54 (62)	67 (72)	64 (67)
S8	55 (66)	55 (62)	57 (68)	63 (72)
S9	63 (65)	61 (74)	62 (68)	61 (71)
S10	66 (70)	71 (65)	74 (77)	75 (91)
S11	63 (64)	65 (67)	65 (68)	61 (62)
S12	73 (79)	75 (84)	77 (84)	79 (84)
S13	62 (71)	62 (72)	64 (71)	63 (74)
S14	59 (62)	66 (71)	67 (65)	63 (69)
S15	50 (46)	50 (53)	50 (52)	55 (59)
S16	51 (55)	67 (77)	68 (72)	75 (77)
S17	54 (62)	59 (54)	62 (71)	66 (78)
S18	66 (64)	61 (53)	67 (75)	65 (72)
Mean	62 (65)	66 (68)	68 (73)	68 **(76)**

**Table II:** The obtained Sen and SPC Values in the Inter-Subject Classification Approach and the Number of Parameters of Each Network. The Values are in Percent (%).

Criteria	LSR [24]	AAE [21]	ASAE [21]	GAN
SEN	61	66	**67**	65
SPC	59	65	68	**70**
# of Parameters	-	3.12×10^6^	3.90×10^6^	**0.25** ×**10^6^**
